# Prevalence of Drug-Resistant Tuberculosis in HIV-Positive and Diabetic Patients in Sinaloa, Mexico: A Retrospective Cross-Sectional Study

**DOI:** 10.3390/tropicalmed9040089

**Published:** 2024-04-22

**Authors:** Analy Aispuro Pérez, Ulises Osuna-Martínez, Jose Angel Espinoza-Gallardo, Luis Alfredo Dorantes-Álvarez, Gerardo Kenny Inzunza-Leyva, Kimberly Estefania Dorantes-Bernal, Geovanna Nallely Quiñonez-Bastidas

**Affiliations:** 1 Facultad de Ciencias Químico-Biológicas, Universidad Autónoma de Sinaloa, Ciudad Universitaria, Culiacan 80013, Sinaloa, Mexico; est.analyap@uas.edu.mx (A.A.P.); ulises.osuna@uas.edu.mx (U.O.-M.); 2 Coordinación Estatal de Tuberculosis, Servicios de Salud de Sinaloa, Secretaria de Salud Blvd, Alfonso Zaragoza Maytorena No. 2204, Fraccionamiento Bonanzas, Culiacan 80020, Sinaloa, Mexicoluis.dorantes@imss.gob.mx (L.A.D.-Á.); 14575663.dorantes@ms.uas.edu.mx (K.E.D.-B.); 3 Centro de Investigación y Docencia en Ciencias de la Salud, Universidad Autónoma de Sinaloa, Eustaquio Buelna 91, Burocrata, Culiacan 80030, Sinaloa, Mexico

**Keywords:** drug resistance, prevalence, tuberculosis, HIV, T2DM

## Abstract

Tuberculosis (TB) is a disease caused by the bacillus *Mycobacterium tuberculosis* (MTB). Human immunodeficiency virus (HIV) infection and type 2 diabetes mellitus (T2DM) are among the main risk factors for the development of TB and increase the risk of drug-resistant TB developing (DR-TB). The aim of this study was to estimate the prevalence of DR-TB in patients with HIV or T2DM in Sinaloa, Mexico. This was an observational and cross-sectional study. The analysis was conducted using the clinical data of patients registered on the National Epidemiological Surveillance System for TB (SINAVE/PUI-TB) platform with a presumed diagnosis of TB during 2019 to 2021 in Sinaloa, Mexico. The prevalence of DR-TB was estimated in HIV and T2DM patients, as well as the odds ratios for their sociodemographic variables, using the Chi-square test. There were 2, 4, and 4 TB-HIV cases and 2, 6, and 9 TB-T2DM cases during 2019, 2020, and 2021, respectively, whereas there were 2 and 1 DRTB-HIV and DRTB-T2DM cases, respectively. The results indicated that the WHO guidelines for DR-TB were not properly applied to this high-risk population. Hence, the appropriate application of guidelines for TB and DR-TB detection in these patients needs to be immediately implemented by the State health system.

## 1. Introduction

According to the 2021 Global Tuberculosis Report, Brazil, Peru, Mexico, Haiti, and Colombia are the countries with the highest incidence of tuberculosis (TB) in Latin America [1]; however, the new 2023 report has showed that TB diagnoses have suffered from a delay in the disease eradication process due to COVID-19 [2]. TB is a disease caused by the bacillus *Mycobacterium tuberculosis* (MTB), with a significant morbidity and mortality risk in adults. Since the emergence of COVID-19, more people have been affected by TB, approximately 9.9 million individuals worldwide: 5.5 million men, 3.3 million women, and 1.1 million children [3,4]. Currently, TB is present across all countries and age groups. Age, gender, delayed disease detection, alcohol, and drug abuse are considered risk factors for the development of TB. Additionally, the presence of other comorbidities such as HIV, T2DM, and drug-resistant (DR) MTB contribute to increased mortality in patients with TB [5,6].

Moreover, Rifampicin (RIF)-resistant MTB strains have significantly complicated TB prevention, control, and treatment, resulting in a health crisis [7]. Traditionally, phenotypic tests like bacilloscopy and bacteriological cultures have been used for TB’s detection [8,9]. However, molecular technology has improved TB diagnosis; the GeneXpert method has allowed for a faster, more sensitive, and specific diagnosis of TB and DR-TB, which in turn has helped in their pharmacological treatment and outcome in patients [10].

The GeneXpert MTB/RIF (GeneXpert) test has generated a significant advance in TB’s molecular diagnosis. Developed by Cepheid Inc., the GeneXpert assay kit is specifically designed for the GeneXpert instrument [11]. This technique utilizes multiplexed semi-nested real-time PCR with fluorescence quantification to detect the MTB rifampicin-resistance determining region, the rpoB gene [12].

Since the WHO implemented its unified guidelines for TB and DR-TB, it has been recommending the use of GeneXpert as the primary diagnostic method for HIV or diabetic patients with suspected TB, as well as for other high-risk populations [13]. In Mexico, there is a national strategy to prevent DR-TB, which includes the selection of the most appropriate initial therapy for TB patients and, specifically, for those whose DR was detected by GeneXpert or conventional methods. Regular surveillance or surveys of the DR of new TB patients are also recommended to monitor resistant trends [12].

Regarding DR-TB’s prevalence in Mexico, in 1997, Granich et al. conducted a population-based study on TB, reporting moderate levels of DR in the states of Baja California, Oaxaca, and Sinaloa (7.9, 12.7, and 8.9%, respectively) [14].

Sinaloa has been identified as an endemic area for TB. Nevertheless, the prevalence of DR-TB in the HIV and T2DM population remains unclear.

Socioeconomic conditions such as poverty, overcrowding, and limited access to healthcare services facilitate the spread of TB within communities, while high-density populations increase the risk of transmission due to enhanced contact between individuals. Moreover, Sinaloa’s geographical location and economic activities, such as agriculture and fishing, attract migrants from other regions, potentially introducing different TB strains [15].

Additionally, the region’s drug-trafficking reputation contributes to high rates of incarceration in prisons that often have conditions conducive to TB transmission, including overcrowding and inadequate healthcare [16]. Rural areas of Sinaloa may also lack sufficient healthcare facilities and resources for TB’s diagnosis, treatment, and prevention, leading to delays in TB’s detection and treatment. From 1997 to 2005, a previous study conducted by Zazueta-Beltran et al. determined the prevalence of DR-TB in Sinaloa and concluded that the proportion of the population with DR-TB increased by 1.2% per year, urging for efforts to be made to decrease its overall prevalence in the state [17].

It is well known that DR-TB in HIV [18] or T2DM patients [19] exacerbates their clinical prognosis and the outcome of the disease. Additionally, these factors have a significant impact on treatment cost, hospitalization, quality of life, and the public health system as a whole [20]. Therefore, the aim of this study was to estimate the prevalence of DR-TB in HIV and T2DM patients through a retrospective data analysis of the SINAVE/PUI-TB platform in Sinaloa from 2019 to 2021.

## 2. Materials and Methods

A retrospective cross-sectional study was performed through the analysis of the clinical records of TB patients registered on the SINAVE/PUI-TB platform in Sinaloa from January 2019 to December 2021. The prevalence of TB and DR-TB to RIF-resistant-TB was estimated in the HIV and T2DM groups with a diagnosis confirmed by GeneXpert [21]. Clinical and demographic variables such as sex, age, weight, BMI (body mass index), size, diseases other than HIV or T2DM, the location of the disease, the institution which provided the diagnosis, treatment outcome, smoking, alcoholism, and drug addiction were analyzed, and odds ratios were estimated. Data were analyzed using descriptive and inferential methods. A Chi-square test was conducted to establish statistical significance (*p* < 0.05). All analyses were performed using GraphPad Prism v.9 [22,23].

### 2.1. Data Source

The SINAVE/PUI-TB “https://www.sinave.gob.mx/.mx (accessed on 30 May 2022)” platform, within which the information from all the Institutions of the Mexican National Health System is found, was used as our data source. The platform is exclusively used by authorized health personnel.

### 2.2. Selection Criteria

**Figure 1:**
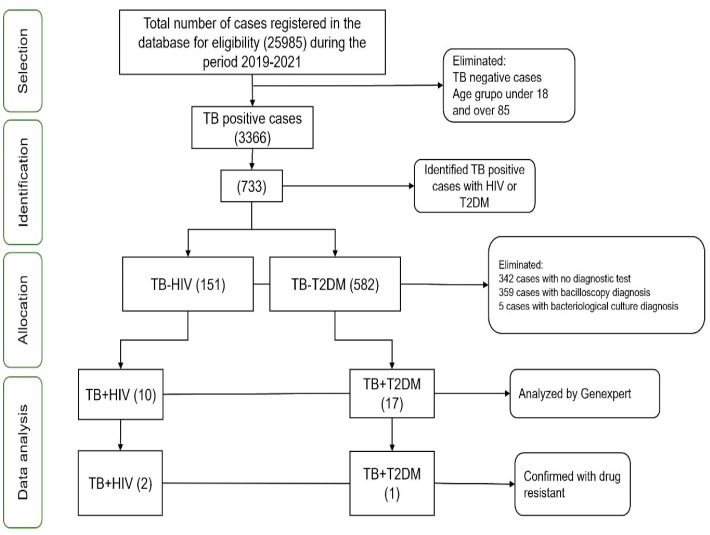
Flowchart of clinical case selection. The data selection process began with a total of 25,985 clinical cases, out of which 733 cases with TB-HIV or TB-T2DM were identified. Among these, 151 cases were assigned to the TB-HIV study group, while 582 cases were assigned to the TB-T2DM study group. Out of these, 27 cases were confirmed by GeneXpert (TB-HIV: 10 and TB-T2DM: 17), with only 3 cases being DR-TB (TB-HIV: 2 and TB-T2DM: 1).

The inclusion criteria were established using the clinical data in SINAVE/PUI-TB platform. The criteria included gender (female or male), a diagnosis established in Sinaloa from 2019 to 2021, an age between 18 and 85, a positive diagnosis for MTB using the GeneXpert technique, and the presence of comorbidities such as HIV or T2DM (Figure 1).

### 2.3. Ethical Approval Declarations

This project was approved by the ethics and research committees of the General Hospital of Culiacan, “Dr. Bernardo J. Gastélum” with number 00087. Additionally, the study protocol was registered in the ISRCTN registry (ISRCTN18957388), https://doi.org/10.1186/ISRCTN18957388).

## 3. Results

### 3.1. Estimation of the Prevalence of TB and DR-TB in Patients with HIV or T2DM by GeneXpert and Bacteriological Diagnoses

A total of 25,985 cases registered in the SINAVE/PUI-TB platform was reviewed; 3366 cases were TB-positive during 2019–2021. Furthermore, 733 cases were HIV- or T2DM-positive, which were assigned to the TB-HIV or TB-T2DM groups (151 for TB-HIV and 582 for TB-T2DM). After these groups were assigned, 27 cases were found to be diagnosed by GeneXpert (TB-HIV: 10 and TB-T2DM: 17). Moreover, 3 cases presented DR-TB (TB-HIV: 2 and TB-T2DM: 1) (Figure 1). Table 1 shows the GeneXpert and bacilloscopy diagnosis data of the TB-HIV and TB-T2DM groups. The percentage of the TB-HIV group that received a GeneXpert diagnosis was 3%, 13.3%, and 7.1% (2, 4, and 4 positive cases); for the TB-T2DM group it was 0.9%, 3.9%, and 4.2%, (2, 6, and 9 positive cases) in 2019, 2020, and 2021, respectively. The TB-positive cases confirmed by bacilloscopy or bacteriological culture were also analyzed. The results showed a total of 36 and 323 bacilloscopy-positive cases for TB-HIV and TB-T2DM, respectively. The prevalence of TB-HIV was 26.1%, 23.3%, and 21.4% (17, 7, and 12 positive cases); whereas for TB-T2DM was 57.2%, 50.6%, and 57.1% (126, 77, and 120 positive cases) from 2019 to 2021. According to bacteriological culture diagnoses, the prevalence of TB-HIV was 3% (2 cases) and 0.4% for TB-T2DM (1 case) in 2019. No cases were reported in 2020 and TB-T2DM’s prevalence was 0.9% (2 cases) in 2021. For all diagnosis methods, their prevalence was estimated from the total positive cases registered in the platform per year for each group (65, 30, and 56 [n = 151] for HIV; 220, 152, and 210 [n = 582] for T2DM). Despite this, in 103 TB-HIV and 239 TB-T2DM cases the diagnosis method was not indicated, which suggests a gap in the management of data on the platform (Figure 1).

DR-TB’s prevalence was estimated according to our research objectives. However, during 2019 and 2021, no DR-TB cases were reported using GeneXpert. Additionally, in 2020, the prevalence was 50% for DRTB-HIV (2 cases) and 16.6% for DRTB-T2DM (1 case). In an extra analysis, three DR-TB records were found; however, their confirmation was by bacilloscopy and subsequent treatment failure, as were 1 case of DRTB-HIV during 2020 and 2 cases of DRTB-T2DM during 2019 (Table 1).

In addition to these results, we analyzed the prevalence of TB-HIV and TB-T2DM cases without drug resistance, obtaining prevalences of 5.6%, 3.1%, and 4.4%, respectively, per year analyzed for the TB-HIV group and a prevalence of 19.0%, 16.1%, and 16.5%, respectively, per year analyzed for the TB-T2DM group (Table 2).

Once the prevalence of patients without drug resistance was determined, a screen was carried out considering the different diagnostic techniques used, obtaining prevalences of 3, 13.3, and 0.07 for the TB-HIV group and 0.9, 0.03, and 0.04 for the TB-T2DM group, per year analyzed, using the GeneXpert diagnostic method. However, the prevalence was higher in patients diagnosed by bacilloscopy, for which prevalences of 16.1, 23.3, and 21.4 were obtained for the TB-HIV group and 57.2, 50.6, and 57.1 for the TB-T2DM group, per year analyzed (Table 1). Furthermore, the sociodemographic quantitative and qualitative characteristics of these patients were also analyzed (Table 3 and Table 4).

### 3.2. Usage Frequency Estimation of the GeneXpert Technique

The usage frequency of the GeneXpert and bacilloscopy techniques was also determined. From the 3366 TB-positive cases registered in the SINAVE/PUI-TB platform, the usage frequency of the GeneXpert technique was 5.8%, in contrast with the 92.26% use of the bacilloscopy method and the 1.94% use of bacteriological cultures (Table 5).

### 3.3. Analysis of the Sociodemographic Characteristics of Patients with TB-DR

Table 6 shows the association between HIV or T2DM with DR-TB and the sociodemographic characteristics of these patients diagnosed in 2020. During 2019 or 2021, no such associations were observed, due to the absence of data on DR-TB diagnosed by GeneXpert. Our analysis of the clinical and demographic variables showed that DR occurs mostly in male cases (2 cases vs. 1 female case) with TB-HIV. On the other hand, in TB-T2DM group, only 1 female DR case was reported. Also, in Table 6, the age, body weight and BMI of the DRTB-HIV and DRTB-T2DM cases are described. With respect to the location of the disease, all registered cases occurred as pulmonary DR-TB.

## 4. Discussion

Despite efforts to control TB, it continues to be one of the major public health problems worldwide [24,25]. In recent years, cases of TB and DR-TB have been increasing, particularly in developing countries. Kampala City, Uganda, reported an increased TB prevalence of 7% [26]; the DR to RIF and isoniazid ratio was 14.25% in Ethiopia, suggesting an increase compared with previous reports [27]. Additionally, epidemiological reports estimated a 11.6% global prevalence of DR-TB, alerting authorities to a wider spread of TB and a potential risk of an increase in mortality [28].

TB is a current health problem in Sinaloa. The most recent epidemiological report in Mexico (2021) indicates that Sinaloa is the place with the second highest incidence rate of TB (38.7) in the country [29]. In Mexico, the most frequent diseases associated with TB are diabetes (20%), malnutrition (13%), HIV/AIDS (10%) and alcoholism (6%) [12].

Authors should discuss their results, and how they can be interpreted from the perspective of previous studies, and their working hypotheses. The findings and their implications should be discussed in the broadest context possible. Future research directions may also be highlighted.

To our knowledge, this is the first report about TB and DR-TB’s prevalence in HIV and T2DM patients in Sinaloa. According to the results, the number of TB-positive cases was lower in patients with HIV (10 cases, during 2019–2021) and T2DM (17 cases during 2019–2021) patients when their TB diagnosis was made using the GeneXpert Technique compared to other diagnostic methods. Consequently, one limitation when determining the prevalence in these patients was the lower use of the GeneXpert technique (5.8%), which does not allow these results to be compared with those of other studies. There are no similar studies in our region to compare our results to; therefore, one of the contributions of this study is the establishment of a new diagnosis methodology for TB and DR-TB in high-risk populations. Previous studies carried out by Zazueta-Beltran *et al*. estimated a high prevalence of DR-TB in Sinaloa (34.9%), compared to the lower prevalence obtained by Granich et al. (8.9%) [14,17].

This can be explained by the fact that developing countries may not have the necessary resources to offer appropriate treatment and follow-up care, which can lead to patients abandoning treatment [28].

Moreover, the estimation of TB’s prevalence relies on a wide range of variables, such as the diagnosis method, comorbidities, region, and other factors. A systematic review and meta-analysis indicated that the overall prevalence of TB-HIV in Iran was 14% [30], whereas the use of the GeneXpert assay in HIV patients from Ataye District Hospital in Ethiopia showed a 7.89% prevalence of TB [31].

Moreover, in a cross-sectional study conducted in a diabetic clinic in Tanzania, the prevalence of TB in diabetic patients was different when two different diagnostic methods were applied to the same population. The results suggested that GeneXpert (54.5% prevalence) is a powerful TB diagnostic method compared with other clinical methods (45.5% prevalence) [32]. This notwithstanding, it is estimated that around 15% of TB cases worldwide can be attributed to T2DM [33].

As was mentioned above, the change in TB’s prevalence depends on the region. The Americas reported a prevalence of 19.32% compared with European, Southeast Asia, Western Pacific, Eastern Mediterranean, and African Regions, which had prevalences of 17.31%, 14.62%, 13.59%, 9.61%, and 9.30%, respectively [34].

Regarding the variations in TB’s prevalence between different regions, we decided to estimate the overall prevalence of TB in HIV and T2DM patients by including all types of diagnosis methods. The analysis shows that there is a variation in its prevalence according to the year; TB’s prevalence was 5.2, 3.18, and 4.14 in HIV patients, whereas in T2DM patients its prevalence was higher: 19.03, 16.15, and 16.54 in 2019, 2020, and 2021, respectively. Concerning this, TB’s prevalence in T2DM patients is very similar to the prevalence of TB in the American region.

DR-TB’s prevalence has been estimated in several regions. In Zimbawe, the use of GeneXpert reported a 4% and 14.2% prevalence of DR-TB and RIF-resistant TB in new vs. relapse cases, respectively [35]. Meanwhile, in Botswana, its prevalence was 1.3% and 7.7% [36], and in Rwanda it was 1.4% and 4.9% for new and relapse patients, respectively [37].

The prevalence of new or relapsed DR-TB cases was not estimated in the present study, due this distinction not being possible. In a previous report by Perez-Navarro et al., the prevalence of DR-TB with respect to RIF-resistant TB was estimated in T2DM patients in Veracruz, Mexico [38]. They found a 14% prevalence, compared to the 16.6% prevalence shown in the results of the present study. Both estimations are close; however, different diagnosis methods for DR-TB’s detection were employed [38]. Comparing these estimations with international epidemiological data, the number of DRTB-T2DM cases in Sinaloa, Mexico, was higher (16%) than in Shandong, China (5.8%) [19]. Moreover, DRTB-HIV registers were not found in Mexico, but studies in other countries have reported a prevalence of 13.6%, in Nigeria, and 28.4%, in Haiti [39,40].

To understand these differences, an additional analysis was carried out to estimate the frequency percentage of GeneXpert’s usage. The results indicated a 1.3%, 2.6%, and 5.2% usage of GeneXpert, compared with 46.1%, 54.2%, and 67% for bacilloscopy and 1%, 0.2%, and 1.7% for bacteriological culture methods in 2019, 2020, and 2021, respectively. These percentages were obtained based on the total number of confirmed TB cases, including those confirmed through GeneXpert, bacilloscopy, and bacteriological culture and those for whom a confirmation method had not been assigned on the platform.

According to DR-TB’s prevalence and the frequency of the use of GeneXpert, there is an inadequate application of the WHO’s TB guidelines [41]. Moreover, the Official Mexican Standard 006-SSA2-2013 for the prevention and control of TB recommends a GeneXpert diagnosis of TB for all patients with primary treatment failure and relapse, TB cases with a positive bacilloscopy test after the second or third month of primary treatment, patients with relapses or readmissions due to treatment abandonment, and patients who had a previous diagnosis of HIV or uncontrolled DM [12].

The WHO reported in 2021 that only 38% of TB patients benefit from a molecular test as a confirmatory diagnostic tool in their manual on universal access to rapid tuberculosis diagnostic tests [2]. In our study, we observed a low frequency of GeneXpert’s usage (1.3%, 2.6%, and 5.2% per year, respectively) in Sinaloa, Mexico. These percentages do not align with the results reported by the WHO. A study published in 2023, which was conducted in Cameroon from 2020 to 2022, concluded that the utilization of molecular techniques enhances TB’s detection in resource-limited areas [42]. Our study supports this by highlighting the economic barriers that must be overcome in the diagnosis of this disease. We invited healthcare systems to conduct a cost analysis of TB’s diagnosis compared to the cost of its misdiagnosis and inadequate treatment (MXN 13 billion annually) [43]. This underscores the importance of considering cost-effectiveness when evaluating diagnostic strategies and emphasizes the need for improved access to molecular diagnostic tools, particularly in regions facing economic constraints.

Despite recommendations and its high sensitivity (95%) and specificity (98%) for detecting MTB and the mutation of the rpoB gene linked to DR to RIF resistance [44], the GeneXpert TB diagnostic tool’s usage is lower compared with that of bacilloscopy in Sinaloa.

Furthermore, this study revealed significant associations between specific risk factors and DR-TB’s prevalence. The risk of DR-TB was found to be two times higher in HIV patients compared to those with T2DM. Recently, in a cross-sectional study in Haiti, scholars reported that DR-TB is 2.5 times more frequent in patients with HIV than in non-HIV patients [40]. Interestingly, no significant associations between T2DM and DR-TB were observed, despite previous reports having suggested a 3.1 times greater risk of TB in T2DM patients when compared to those without T2DM [45]. This discrepancy aligns with the findings from a cohort study in Barcelona, which indicated a higher risk of developing TB in diabetic patients (HR 1.77) [46]. Otherwise, a systematic review and meta-analysis underscore the high global incidence and prevalence of TB among T2DM patients, emphasizing the urgent need for preventive interventions, particularly in countries with a high TB burden [46].

On the other hand, sociodemographic factors, including age, gender, and lifestyle, such as alcoholism and smoking, as well as specific immunological conditions like HIV and T2DM and substandard housing conditions, play pivotal roles in TB’s development [47,48]. Related to this, Timire et al. identified the key risk factors for DR-TB and RIF-resistant TB, which encompass a history of prior TB treatment, a self-reported HIV infection, and an age under 15 [35]. Furthermore, specific clinical characteristics in diabetic patients, such as an older age, higher BMI, and elevated HbA1C levels, were associated with the prevalence of TB in diabetic patients and correlated with the severity of their TB [49].

Reports in the literature indicate that the adaptive immune response in HIV patients is affected due to their decrease in CD4+ T cells and macrophages (this mechanism was evaluated in an in vivo model). The authors conclude that CD4+ T cell depletion due to HIV infection plays a significant role in increasing the risk of TB [50].

On the other hand, in 2019, Martinez et al. reported that, in patients with T2DM, their adaptive immune response is deficient due to the impaired recruitment and function of their antigen-presenting cells, resulting in a decrease in the Th1, Th2, and Th17 cells that play an important role in macrophage activation and the inflammatory response to TB [51].

Our findings emphasize the need to improve the strategies focused on the clinical management of co-infected patients with TB-HIV or TB-T2DM. Moreover, our results showed two male DRTB-HIV cases and one female DR-TB-T2DM case. It has been described that TB and DR-TB affect males to a greater extent [52]. Labor activities are linked to the risk to TB infection, as are a poor follow-up of the disease and treatment abandonment; these factors are associated with its high prevalence in males. The national survey of epidemiology in Spain (2021) reported that 63% of all TB infections occurred in men [53]. Additionally, the WHO reports highlight that individuals infected with HIV are 18 times more susceptible to developing active TB, and this risk is also elevated in individuals with other immune-compromising disorders, such as T2DM [54]. Finally, the major limitation of this study was that the incomplete data collected by different healthcare personnel may lack important details necessary for a comprehensive analysis of TB cases, which could lead to selection bias. Additionally, the variability in documentation practices between providers and confounding factors not initially considered may complicate the study results. Moreover, loss at follow-up due to missing records could introduce bias, and there is concern regarding the study’s limited generalizability beyond the specific population and time period studied. Furthermore, temporal trends in healthcare practices or diagnostic criteria may also affect the results’ interpretation. Lastly, concerns may arise about the accuracy and consistency of the clinical cases captured if there is no direct supervision during data collection. However, as a whole, the results suggest a high prevalence of DR-TB in two high-risk populations, HIV and T2DM patients.

Currently, in Sinaloa, there are TB programs such as the Tuberculosis Prevention and Control Program [55], which aims to contribute to the well-being of the population by reducing the health damages caused by mycobacterial diseases (TB and leprosy) through prevention and comprehensive care. It also aims to strengthen the implementation of the Strictly Supervised Shortened Treatment Strategy (TAES) by conducting supervisory visits and providing advice to health units, consolidating the National Strategy for the Care of Multidrug-Resistant Tuberculosis Cases, and improving comprehensive care for TB-HIV and TB-DM comorbidities [55].

## 5. Conclusions

Despite the objective of the present study being to estimate the prevalence of drug-resistant TB in HIV and T2DM patients, we found that the number of cases diagnosed using the appropriate GeneXpert methodology was low. A total of 157 and 582 positive TB cases were found in HIV and T2DM patients out of the total 3366 TB-positive cases reported in Sinaloa’s population during 2019 to 2021. Furthermore, only 10 and 17 cases of HIV and T2DM patients with TB were diagnosed by the GeneXpert method, of which only 2 and 1 cases were drug-resistant. Taken together, the data suggest that no drug resistance could be observed in this study population due to the low use of the GeneXpert technique (5.8%). This data analysis indicates that the WHO and Mexican guidelines for the diagnosis of DR-TB were not implemented properly in HIV or T2DM patients; hence, this cross-sectional study recommends that the health system improve the management of the SINAVE/PUI-TB platform, as well as the application of GeneXpert for DR-TB’s detection in HIV and T2DM patients. A suggestion from the present study is to invite those responsible for these programs to periodically review the databases that contain the diagnoses of this disease. By continually monitoring the data, program managers can identify trends, gaps, and areas for improvement in TB’s detection and treatment. This proactive approach allows for timely interventions and adjustments to strategies, ultimately leading to better outcomes for TB patients and reducing the burden of the disease in the community. Additionally, periodic reviews of the databases foster accountability and transparency in program management, improving public trust and support for anti-TB initiatives. Our work offers constructive criticism and extends an invitation for healthcare services to improve state TB programs.

## Figures and Tables

**Table 1:** Prevalence of TB and drug-resistant TB identified by GeneXpert, bacilloscopy, and bacteriological cultures.

Year	2019		2020		2021		Number of Smears	Treatment Failure
Study Groups	Cases	Prevalence (%)	Cases	Prevalence (%)	Cases	Prevalence (%)		
GeneXpert								
TB-HIV	2	3	4	13.3	4	7.1	-	-
TB-T2DM	2	0.9	6	3.9	9	4.2	-	-
Bacilloscopy								
TB-HIV	17	26.1	7	23.3	12	21.4	-	-
TB-T2DM	126	57.2	77	50.6	120	57.1	-	-
Bacteriological culture								
TB-HIV	2	3	-	-	-	-	-	-
TB-T2DM	1	0.4	-	-	2	0.9	-	-
Unestablished diagnostic method								
TB-HIV	44	67.6	19	63.3	40	71.4	-	-
TB-T2DM	91	41.3	69	45.3	79	37.6	-	-
GeneXpert								
DR-TB-HIV	0	-	2	50	0	-	-	-
DR-TB-T2DM	0	-	1	16.6	0	-	-	-
Bacilloscopy								
DRTB-HIV	0	-	1	-	0	-	2	Relapse
DRTB-T2DM	1	-	0	-	0	-	2	Re-entry
	1	-	0	-	0	-	4	Relapse

TB: tuberculosis; DR-TB: drug-resistant tuberculosis; HIV: human immunodeficiency virus; T2DM: type 2 diabetes mel.

**Table 2:** Prevalence of TB with HIV or T2DM registered on the SINAVE platform.

Year	2019		2020		2021	
Groups	Cases	Prevalence	Cases	Prevalence	Cases	Prevalence
TB-HIV	65	5.62	30	3.18	56	4.41
TB-T2DM	220	19.03	152	16.15	210	16.54

**Table 3:** Quantitative sociodemographic characteristics of TB-HIV and TB-T2DM patients.

Year	2019				2020				2021			
Groups	HIV	SD	T2DM	SD	HIV	SD	T2DM	SD	HIV	SD	T2DM	SD
Male												
Cases	58		115		29		97		53		134	
Weight	60.96	17.70	67.65	11.87	60.39	11.15	70.20	16.47	62.17	11.25	69.96	19.81
Size	166.10	20.56	162.55	34.70	169.17	7.00	164.21	27.23	170.09	7.15	166.81	17.75
BMI	22.26	8.76	24.41	3.55	18.93	1.33	24.05	11.59	21.50	3.66	24.16	5.34
Age	37.22	12.04	52.00	13.10	35.03	8.26	51.58	14.07	39.79	12.37	50.52	15.45
Female												
Cases	5		101		1		53		4		82	
Weight	50.60	10.43	63.70	16.05	49.00	N/A	59.90	12.37	51.37	13.57	63.28	14.24
Size	155.20	9.36	157.86	8.50	162.00	N/A	151.17	29.10	158.25	7.13	157.25	8.20
BMI	21.91	6.57	33.78	50.06	18.67	N/A	24.36	2.89	20.31	4.03	25.55	5.36
Age	37.00	6.89	53.72	12.53	28.00	N/A	55.25	12.37	32.50	16.25	54.43	14.13

The values are the frequencies found in the data. HIV: human immunodeficiency virus; T2DM: type 2 diabetes mellitus; BMI: body mass index. N/A: it was not possible to obtain this result, SD: standard deviation.

**Table 4:** Qualitative sociodemographic characteristics of TB-HIV and TB-T2DM patients.

Year	2019 (n = 280)		2020 (n = 180)		2021 (n = 273)	
Group	HIV	T2DM	HIV	T2DM	HIV	T2DM
	(n = 65)	(n = 215)	(n = 27)	(n = 153)	(n = 59)	(n = 214)
^†^ Another comorbidity	AHT: 1	Anemia: 5	Not assigned	AHT: 26	Kaposi sarcoma: 1	AHT: 29
	Chronic hepatitis: 1	AHT: 33		Rheumatoid arthritis: 3	Pulmonary aspergillosis: 1	Anemia: 2
	Hepatitis C: 2	Hypothyroidism: 3		Others: 3		COPD: 3
		Others: 10				COVID-19: 1
Location	Ganglion: 5	Ganglion: 1	Ganglion: 2	Ganglion: 1	Ganglion: 5	Bone: 2
	Intestinal: 2	Meninges: 1	Meninges: 1	Intestinal: 3	Intestinal: 5	Ganglion: 1
	Meninges: 4	Miliary: 1	Miliary: 3	Meninges: 2	Meninges: 5	Intestinal: 1
	Miliary: 12	Pleural: 10	Pleural: 1	Miliary: 2	Miliary: 6	Meninges: 2
	Pleural: 2	Pulmonary: 198	Pulmonary: 24	Pleural: 5	Pulmonary: 32	Miliary: 6
	Pulmonary: 39	Others: 12	Others: 1	Pulmonary: 140	Others: 4	Pleural: 5
	Others: 2			Others: 2		Pulmonary: 196
						Skin: 2
* Diagnostic method	BAAR: 17	BAAR: 126	BAAR: 7	BAAR: 78	BAAR: 12	BAAR: 123
	Bact. culture: 2	Bact. culture: 1	GeneXpert: 5	GeneXpert: 6	GeneXpert: 4	Bact. culture: 2
	GeneXpert: 2	GeneXpert: 2	Not assigned: 20	Not assigned: 71	Not assigned: 41	GeneXpert: 9
	Not assigned: 44	Not assigned: 91				Not assigned: 81
Smoking	1	13	4	6	6	12
Alcoholism	1	14	4	6	2	8
Drug addiction	6	4	2	4	5	9

AHT: Artery Hypertension, Bact. Culture: bacteriological culture, COPD: Chronic Obstructive Pulmonary Disease, HIV: human immunodeficiency virus; T2DM: type 2 diabetes mellitus. ^†^ In another comorbidity or diagnostic method: the information was not captured by medical staff. * Diagnostic method: the clinical cases analyzed were positive for TB and were confirmed by different methods; however, these cases were not drug-resistant.

**Table 5:** Frequency of usage of GeneXpert, bacilloscopy and bacteriological cultures (2019–2021).

Techniques	Frequency (%)
GeneXpert	5.81
Bacilloscopy	92.26
Bacteriological culture	1.94

**Table 6:** Sociodemographic characteristics and associations with DR-TB clinical cases confirmed by GeneXpert.

Groups of Study	2019 (n = 0)		2020 (n = 3)			2021 (n = 0)		
Characteristics	HIV (n = 0)	T2DM (n = 0)	HIV (n = 2)	OR	T2DM (n = 1)	OR	HIV (n = 0)	T2DM (n = 0)
Sex								
Male	-	-	2 (100%)	2.0	-	-	-	-
Female	-	-	-	-	1 (100%)	0.49	-	-
Age	-	-	36		56		-	-
BMI	-	-	20.41		17.78		-	-
Size	-	-	170		166		-	-
Weight	-	-	59		49		-	-
Another comorbidity	-	-	-		-		-	-
Location	-	-	Pulmonary (100%)		Pulmonary (100%)	-		-
Institution that provides care	-	-	SSA (100%)		SSA (100%)		-	-
Diagnostic method	-	-	GeneXpert (100%)		GeneXpert (100%)		-	-
Smoking	-	-	-		-		-	-
Alcoholism	-	-	-		-		-	-
Drug addiction	-	-	-		-		-	-

The values are the frequencies found in the data. HIV: human immunodeficiency virus; T2DM: type 2 diabetes mellitus; OR: odds ratio; SSA: secretary of health; BMI: body mass index.

## Data Availability

The datasets generated and/or analyzed during the current study are not publicly available due to confidential information being contained on the platform, including patients’ personal data, but they are available from the corresponding author upon reasonable request.

## References

[1] (2021). Global Tuberculosis Report 2021. https://www.ptonline.com/articles/how-to-get-better-mfi-results.

[2] (2023). Global Tuberculosis Report 2023. https://iris.who.int/.

[3] The End of Tuberculosis. https://iris.who.int/bitstream/handle/10665/331326/WHO-HTM-TB-2015.19-eng.pdf?sequence=1.

[4] Tuberculosis. https://www.who.int/news-room/fact-sheets/detail/tuberculosis.

[5] Alejandra N.C.D., Ezequiel R.B.M. (2020). Factores asociados a la prevalencia de tuberculosis en la Jurisdicción Sanitaria 3, La Paz, Baja California Sur. Avan Cien Sal Med..

[6] Basic TB Facts|TB|CDC. https://www.cdc.gov/tb/topic/basics/default.htm#print.

[7] Gómez-Tangarife V.J., Gómez-Restrepo A.J., Robledo-Restrepo J., Hernández-Sarmiento J.M. (2018). Drug resistance in mycobacterium tuberculosis: Contribution of constituent and acquired mechanisms. Rev. Salud Publica.

[8] Campelo T.A., Cardoso de Sousa P.R., Nogueira L.d.L., Frota C.C., Zuquim Antas P.R. (2021). Revisiting the methods for detecting Mycobacterium tuberculosis: What has the new millennium brought thus far?. Access Microbiol..

[9] Gholoobi A., Masoudi-Kazemabad A., Meshkat M., Meshkat Z. (2014). Comparison of Culture and PCR Methods for Diagnosis of Mycobacterium tuberculosis in Different Clinical Specimens. Jundishapur J. Microbiol..

[10] Tamirat K.S., Kebede F.B., Baraki A.G., Akalu T.Y. (2022). The Role of GeneXpert MTB/RIF in Reducing Treatment Delay Among Multidrug Resistance Tuberculosis Patients: A Propensity Score Matched Analysis. Infect. Drug Resist..

[11] MANUAL OPERATIVO Implementación del GeneXpert MTB/RIF en el Programa de Tuberculosis. https://diprece.minsal.cl/wrdprss_minsal/wp-content/uploads/2018/02/2018.01.23_MANUAL-XPERT.pdf.

[12] PC, SPPS SECRETARIA DE SALUD NORMA Oficial Mexicana NOM-006-SSA2-2013, Para la Prevención y Control de la Tuberculosis. https://www.gob.mx/cms/uploads/attachment/file/10390/NOM-006-SSA2-2013.pdf.

[13] World Health Organization (2020). WHO Consolidated Guidelines on Tuberculosis. Module 1, Prevention: Tuberculosis Preventive Treatment.

[14] Granich R.M., Balandrano S., Santaella A.J., Binkin N.J., Castro K.G., Marquez-Fiol A., Anzaldo G., Zarate M., Jaimes M.L., Velazquez-Monroy O. (2000). Survey of Drug Resistance of Mycobacterium tuberculosis in 3 Mexican States, 1997. Arch. Intern. Med..

[15] López López R.C. (2020). Emigración forzada de familias por la violencia en el sur de Sinaloa: Experiencias trágicas y complejas. Secuencia.

[16] López R. (2017). Experiencias de emigración forzada de familias por la violencia en Sinaloa 2006–2016. Desplazamiento Interno e Integración Social.

[17] Zazueta-Beltran J., León-Sicairos N., Muro-Amador S., Flores-Gaxiola A., Velazquez-Roman J., Flores-Villaseñor H., Canizalez-Roman A. (2011). Increasing drug resistance of *Mycobacterium tuberculosis* in Sinaloa, Mexico, 1997–2005. Int. J. Infect. Dis..

[18] World Health Organization (WHO) (2014). Companion Handbook to the WHO Guidelines for the Programmatic Management of Drug-Resistant Tuberculosis.

[19] Song W.M., Shao Y., Liu J.Y., Tao N.N., Liu Y., Zhang Q.Y., Xu T.T., Li S.J., Yu C.B., Gao L. (2019). Primary drug resistance among tuberculosis patients with diabetes mellitus: A retrospective study among 7223 cases in China. Infect. Drug Resist..

[20] Hannah H.A., Miramontes R., Gandhi N.R. (2017). Sociodemographic and clinical risk factors associated with tuberculosis mortality in the United States, 2009–2013. Public Health Rep..

[21] Xpert® MTB/RIF. https://www.cepheid.com/es/tests/Critical-Infectious-Diseases/Xpert-MTB-RIF.

[22] D’Arrigo G., Gori M., Pitino A., Tsalikakis D.G., Liakopoulos V., Roumeliotis S., Tripepi G. (2023). Measures of frequency and effect in clinical research. International Urology and Nephrology.

[23] Ferrer M.E.F., Del Prado González N. (2013). Medidas de frecuencia y de asociación en epidemiología clínica. An. Pediatría Contin..

[24] Tuberculosis—OPS/OMS|Organización Panamericana de la Salud. https://www.paho.org/es/temas/tuberculosis.

[25] Espinal M.A., Laszlo A., Simonsen L., Boulahbal F., Kim S.J., Reniero A., Hoffner S., Rieder H.L., Binkin N., Dye C. (2001). Global trends in resistance to antituberculosis drugs. World Health Organization-International Union against Tuberculosis and Lung Disease Working Group on Anti-Tuberculosis Drug Resistance Surveillance. N. Engl. J. Med..

[26] Izudi J., Bajunirwe F., Cattamanchi A. (2023). Increase in rifampicin resistance among people previously treated for TB. Public Health Action.

[27] Reta M.A., Tamene B.A., Abate B.B., Mensah E., Maningi N.E., Fourie P.B. (2022). Mycobacterium tuberculosis Drug Resistance in Ethiopia: An Updated Systematic Review and Meta-Analysis. Trop. Med. Infect Dis..

[28] Salari N., Kanjoori A.H., Hosseinian-Far A., Hasheminezhad R., Mansouri K., Mohammadi M. (2023). Global prevalence of drug-resistant tuberculosis: A systematic review and meta-analysis. Infect. Dis. Poverty.

[29] SITUACIÓN EPIDEMIOLÓGICA MÉXICO 2021. https://www.gob.mx/salud/documentos/boletinepidemiologico-sistema-nacional-de-vigilancia-epidemiologica-sistema-unico-de-informacion-261547.

[30] Pourakbari B., Mamishi S., Banar M., Keshtkar A.A., Mahmoudi S. (2019). Prevalence of TB/HIV co-infection in Iran: A systematic review and meta-analysis. Ann. Ig..

[31] Gebretsadik D., Ahmed N., Kebede E., Mohammed M., Belete M.A. (2020). Prevalence of Tuberculosis by Automated GeneXpert Rifampicin Assay and Associated Risk Factors Among Presumptive Pulmonary Tuberculosis Patients at Ataye District Hospital, North East Ethiopia. Infect. Drug Resist..

[32] Makuka G.J., Balandya E., Munseri P. (2022). Original research: Burden of active pulmonary tuberculosis among patients with diabetes in Dar es Salaam, Tanzania: A cross-sectional study. BMJ Open.

[33] Kansal H.M.L., Srivastava S., Bhargava S.K. (2021). Diabetes Mellitus and Tuberculosis. J. Int. Med. Sci. Acad..

[34] Li M., Chen T., Hua Z., Yan H., Wang D., Li Z., Kang Y., Zhu N., Li C. (2021). Global, regional, and national prevalence of diabetes mellitus in patients with pulmonary tuberculosis: A systematic review and meta-analysis. Diabetol. Metab. Syndr..

[35] Timire C., Metcalfe J.Z., Chirenda J., Scholten J.N., Manyame-Murwira B., Ngwenya M., Matambo R., Charambira K., Mutunzi H., Kalisvaart N. (2019). Prevalence of drug-resistant tuberculosis in Zimbabwe: A health facility-based cross-sectional survey. Int. J. Infect. Dis..

[36] Tembo B.P., Malangu N.G. (2019). Prevalence and factors associated with multidrug/rifampicin resistant tuberculosis among suspected drug resistant tuberculosis patients in Botswana. BMC Infect. Dis..

[37] Habimana-Mucyo Y., Dushime A., Migambi P., Habiyambere I., Semuto Ngabonziza J.C., Decroo T. (2023). Continuous surveillance of drug-resistant TB burden in Rwanda: A retrospective cross-sectional study. Int. Health.

[38] Perez-Navarro L.M., Restrepo B.I., Fuentes-Dominguez F.J., Duggirala R., Morales-Romero J., López-Alvarenga J.C., Comas i., Zenteno-Cuevas R. (2017). The effect size of type 2 diabetes mellitus on tuberculosis drug resistance and adverse treatment outcomes. Tuberculosis.

[39] Ugwu K.O., Agbo M.C., Ezeonu I.M. (2021). Prevalence of Tuberculosis, Drug-Resistant Tuberculosis and HIV/TB Co-Infection in Enugu, Nigeria. Afr. J. Infect. Dis..

[40] Hoffmann J., Chedid C., Ocheretina O., Masetti C., Joseph P., Mabou M.M., Mathon J.E., Francois E.M., Gebelin J., Babin F.X. (2021). Drug-resistant TB prevalence study in 5 health institutions in Haiti. PLoS ONE.

[41] (2020). WHO Consolidated Guidelines on Tuberculosis. WHO Consolidated Guidelines on Tuberculosis: Module 4: Treatment—Drug-Resistant Tuberculosis Treatment. https://www.ncbi.nlm.nih.gov/books/NBK558570/.

[42] Vuchas C., Teyim P., Dang B.F., Neh A., Keugni L., Che M., Che P.N., Beloko H., Fondoh V., Ndi N.N. (2023). Implementation of large-scale pooled testing to increase rapid molecular diagnostic test coverage for tuberculosis: A retrospective evaluation. Sci Rep..

[43] Organización Mundial de la Salud (OMS) (2021). PAG WEB. https://www.who.int/es/news-room/fact-sheets/detail/tuberculosis.

[44] (2020). Trademark, Patents and Copyright Statements. https://www.cepheid.com/content/dam/www-cepheid-com/documents/package-insert-files/Xpert-MTB-RIF-SPANISH-Package-Insert-301-1404-ES-Rev-G.pdf.

[45] Kumar Nathella P., Babu S. (2017). Influence of diabetes mellitus on immunity to human tuberculosis. Immunology.

[46] Antonio-Arques V., Franch-Nadal J., Caylà J.A. (2021). Diabetes y tuberculosis: Una sindemia complicada por la COVID-19. Med. Clin..

[47] Medina A., López L., Martínez C., Aguirre S., Alarcón E. (2019). Factors associated with tuberculosis mortality in Paraguay, 2015–2016. Rev. Panam. Salud Publica/Pan Am. J. Public Health.

[48] Luiz-Tornero A.M., Sánchez-Recio R. (2022). Tuberculosis y factores socioeconómicos en la población española: Una revisión sistemática. Rev. Esp. Salud. Pública.

[49] Ugarte-Gil C., Alisjahbana B., Ronacher K., Riza A.L., Koesoemadinata R.C., Malherbe S.T., Cioboata R., Llontop J.C., Kleynhans L., Lopez S. (2020). Diabetes Mellitus Among Pulmonary Tuberculosis Patients From 4 Tuberculosis-endemic Countries: The TANDEM Study. Clin. Infect. Dis..

[50] Bell L.C.K., Noursadeghi M. (2018). Pathogenesis of HIV-1 and mycobacterium tuberculosis co-infection. Nat. Rev. Microbiol..

[51] Martinez N., Kornfeld H. (2019). Tuberculosis and diabetes: From bench to bedside and back. Int. J. Tuberc. Lung Dis..

[52] Informe Técnico Igualdad de Género. https://www.theglobalfund.org/media/5729/core_gender_infonote_es.pdf.

[53] Vigilancia de la Tuberculosis. Año 2022 Resultados de la Red Nacional de Vigilancia Epidemiológica. https://www.isciii.es/QueHacemos/Servicios/VigilanciaSaludPublicaRENAVE/EnfermedadesTransmisibles/Documents/archivos%20A-Z/Tuberculosis/RENAVE_informe_Vigilancia%20TB_%202022.pdf.

[54] TB and HIV Coinfection|TB|CDC. https://www.cdc.gov/tb/topic/basics/tbhivcoinfection.htm.

[55] Programa Prevención y Control de la Tuberculosis. https://saludsinaloa.gob.mx/index.php/programa-de-prevencion-y-control-de-la-tuberculosis/.

